# Supplementary material for: Are carbon emissions trading and green financial instruments synergistic? -Comprehensive quantitative research based on content analysis
Source: PLoS One. 2024 Mar 7;19(3):e0298601. doi: 10.1371/journal.pone.0298601 (PMC10919601; doi:10.1371/journal.pone.0298601)
Supplement: S1 Data — (ZIP) [file pone.0298601.s001.zip › Data/地级市Z_F工作报告.zip!/╣π╢1⁄2/╢1⁄2▌╕╩╨/└┤╫╘╣1⁄2╓┌║┼í╛┬φ┐╦╩2╛▌═°í┐_2011.docx]

各位代表：

现在，我代表市人民政府向大会作政府工作报告，请予审议，并请政协各位委员和其他列席人员提出意见。

2010年及“十一五”时期工作回顾

刚刚过去的2010年，是我市继续巩固应对国际金融危机成果、加快转变经济发展方式的重要一年。面对有所好转但仍然复杂的外部形势，面对繁重艰巨的转型发展任务，面对深层矛盾日益凸显、利益深刻变动的社会格局，我们在上级和市委的坚强领导下，以科学发展观统领全局，全面实施珠三角规划纲要，突出以转变发展方式为主线，继续稳增长，加力调结构，积极优环境，全面强管理，锐意抓改革，着力保民生，推动经济社会在平稳发展的基础上加快转型，取得了扎实成效。东莞成为全国加工贸易转型升级试点城市，获评中国全面小康特别贡献城市、中国十佳绿色城市、中国十大最关爱民生的城市，创建国家环保模范城市通过了国家验收。具体体现在以下六个方面：

一、力保平稳增长，切实巩固了经济回升势头。紧紧抓住外部环境有所好转的时机，深入实施刺激经济增长的一系列政策措施，巩固应对金融危机的成果，保持了经济平稳较快增长。预计全市实现生产总值4246亿元，比上年增长10.3%，人均生产总值66344元，增长14.6%。来源于东莞的财政总收入785亿元，增长25.1%，其中市财政一般预算收入278亿元，增长20.2%。金融机构本外币各项存款余额6078亿元，比年初增长19.3%。坚持不懈帮扶企业。认真落实“六个10亿元”政策，加强用工、用电保障和融资、通关等服务，加快发展保税物流，开展人民币贸易结算试点业务。企业经营明显好转，外资企业关停外迁数比上年减少11%，增资扩产617宗，增加133宗。工业生产和出口均超过危机前的水平，全市规模以上工业增加值1813亿元，增长19%；进出口总额1213亿美元，增长28.8%，其中出口696亿美元，增长26.1%。坚持不懈扩大内需。落实领导挂钩督导和审批绿色通道等制度，加快重点基础设施和优质产业项目建设，完成固定资产投资1115亿元。实施八大商贸促进工程，培育和引导消费，社会零售总额1108亿元，增长15.9%。优化外企内销通道，成功举办外博会、台博会、动漫展等，开展莞货展销活动，筹建工厂直销中心，全市工业产品内销总额3537亿元，增长23.6%。坚持不懈扶持镇村。落实财政超收分成、公共管理支出补助、生态补偿等政策，对镇街统筹土地给予借款支持，实施市内“双到”扶贫。全年市财政共安排65亿元支持镇村建设，预计32个镇街可支配收入280.7亿元，村组两级可支配收入183亿元，分别增长14.8%和6.1%。城市居民人均可支配收入36350元，农村居民人均纯收入14254元，分别增长10%和9.1%。

二、加力调整结构，产业转型迈出了坚实步伐。把调整产业结构作为核心任务，推进经济发展方式有效转变。突出抓好外经贸“五个1000”专项工作。加快加工贸易转型升级，支持企业引进先进技术、优化生产模式、拓展内销市场，全年共受理1250家来料加工企业转型，新增外资企业研发机构150个，是历年总数的1.5倍。着力提高利用外资质量，积极组织赴美加、日韩台等地招商，主动拜访外企总部，邀请重点企业来莞考察，全年合同利用外资26亿美元，实际利用外资27.3亿美元，分别增长60.7%和5.3%。突出培育重点企业和新兴产业。认定现代产业体系“四个30项目”、商贸流通业“四个十大”项目、民营工业50强、服务业50强和首批总部企业等，实施重点扶持。推动3家企业成功上市。加快发展战略性新兴产业，薄膜太阳能光伏和物联网2个基地被列为全省战略性新兴产业基地。制定珠三角新兴物流城市发展规划。突出增强自主发展能力。完善科技东莞政策，深化产学研合作，开展质量强市活动，推动“两自”企业发展。全市工业企业技改投资198亿元，增长28.2%，专利授权量增长70%。新增省级产业集群升级示范区2个、国家高新技术企业91家、省民营科技企业126家、省级以上技术工程中心14个、企业博士后科研工作站5个、省级以上名牌名标56个，参与制修订各类技术标准37项，获批省创新科研团队3个。完善和落实民营经济扶持措施，加强内资引进，民营企业登记户数增长3.1%，实际引进内资176.2亿元，增长45.4%。突出加快园区开发建设。松山湖成功晋升国家级高新区，台湾高科技园开园，粤港澳文化创意产业实验园落户，工业产值和税收总额分别增长23.7%和53.9%。虎门港泊位码头建设加快，保税物流中心投入运行，对台直航航线正式通航。东莞生态园基础建设加快，生态修复初见成效，首次对外公开招商。长安新区用海申报、规划编制等工作扎实推进。基本建成7个农业产业园。突出推进节能降耗减排。落实企业主体责任，强化重点企业监管，加快高耗能、高污染企业的治理改造，淘汰落后产能，推广清洁生产，加强土地执法监察和闲置地处置。全市单位生产总值耗地、耗能分别下降8.3%和2%。预计二氧化硫和化学需氧量排放分别下降4.2%和3.7%。

三、统筹规划建设，持续改善了城乡环境。坚持规划先行、城乡统筹，推进环境整治，创建宜居城乡。着力抓好基础设施建设。环莞快速一期等39项市属重点工程竣工，市轨道交通R2线试验段等26项工程动工。完成3个市区内涝点整治和75个欠发达村老化水管改造。建成镇村联网路31条、110千伏及以上输变电工程21项、水利防灾减灾工程71宗、天然气管网131公里。着力抓好城乡环境整治。扎实推进绿道网建设，建成区域绿道225公里。加快森林公园设施配套，植树造林1800多公顷。全面完成在建污水处理厂主体工程，完成运河整治应急工程，全市污水处理能力大大提高，内河涌和水库污染治理有效加强。着力抓好“三旧”改造。出台土地出让金调整、容积率计算、拆迁补偿指引等配套文件，完成30万亩土地的专项规划和标图建库，各镇街编制了5年专项规划和年度实施计划，全市启动改造地块174宗、约3.4万亩。

四、强化社会管理，有效维护了安全稳定。落实综治维稳责任，促进了社会安定有序。加强治安综合治理。严厉打击恶性和多发性犯罪，大力开展扫黄禁赌等专项行动，深入整治治安重点区域和行业，加强巡逻防控与校园安保。新建省“六好”平安和谐社区41个，市平安社区218个，全年刑事案件破案宗数增长26.9%。加强安全防范工作。深入推进危险化学品、“三小”场所、出租屋、建筑安全、食品药品质量等专项整治，开展创新消防管理试点，落实挂牌督办任务，排查和整改了一批安全隐患。新建镇街应急避灾中心17个，建成食品安全样板市场67个，全市各类安全事故宗数、死亡人数分别下降3.2%和2.2%。加强市政交通管理。强化城管综合执法，深入整治城市“六乱”，加强市政设施、环卫绿化等管理养护。完善道路护栏设施和步行系统，完成一批交通隐患点的整治，严查酒后驾驶。发展公共交通，新增公交运力120辆、线路12条，更新出租车600辆，新建了一批首末站和候车亭，建成运河路自行车专用道示范工程。加强矛盾纠纷调解。完成镇街综治信访维稳中心和村（社区）综治工作站的建设，落实领导接访包案和带案下访等制度，群众信访总量下降23.4%，纠纷调处成功率达93%。加强劳动执法监察和劳资关系调解，劳资突发事件、欠薪逃匿案件分别下降3.4%和37 %。

五、办好民生实事，全面发展了社会事业。市财政用于民生事业支出116.4亿元，基本完成向市民承诺的十件实事。切实做好就业培训工作。加强就业服务，提高最低工资标准，推广“村民车间”模式，继续实施免费技能培训、工资差额补贴、岗位津贴等政策，全年共培训劳动力13.1万人次，发放就业补贴2.7亿元，户籍大学应届毕业生就业率96%，城镇登记失业率控制在2%以内。切实提高社会保障水平。两次调整医保待遇，最高支付额提至15万元，职工和农（居）民退休金分别增长10.2%和44.8%。建立低保标准自然增长机制，向困难人群发放临时物价补贴，发放高龄津贴，设立自然灾害人身保障金。加强住房保障工作，帮助2978户低收入家庭解决住房困难。开展“广东扶贫济困日”和首届“东莞慈善日”活动。积极做好残疾人康复、教育、就业服务。切实繁荣文化教育事业。出台文化名城建设规划纲要和若干政策，举办各级各类文化系列活动，新入选3项国家级非物质文化遗产，东莞文艺作品首获文华大奖，东莞作家首获鲁迅文学奖。加快学校布局调整，完成扩建新建6所中学。出台助学贷款和学历进修补助政策。新招收近1.5万名新莞人子女入读公办学校，设立民办学校扶持专项资金。高考每万户籍人口升大学人数居全省首位。切实推动医疗、体育等事业发展。完善社区卫生服务体系，加快市中医院新院等大型医疗机构建设，有效防控基孔肯雅热等疫情。广泛开展群众体育运动，成功承办第16届亚运会举重赛事，参加第13届省运会取得佳绩，宏远男篮第六次夺取全国联赛冠军。优化计生服务，开展第六次全国人口普查，人口自然增长率6.23‰。国防人防、拥军优属、科普法普、外事侨务、海洋渔业、工青妇幼、民族宗教、统计审计、气象测报、档案方志、志愿服务、打私等各项工作扎实推进。

六、深化各项改革，激发了体制机制活力。坚持先行先试，锐意改革创新，加强法治政府建设，提升行政服务效能。深化政府机构改革。完成部门“三定”方案，市政府工作部门从37个调整为32个，市直临时议事协调机构精简53%。深化事业单位改革。实施事业单位岗位设置和人员聘用制度，新进人员实行公开招考。深化镇村管理体制改革。推进石龙、塘厦简政强镇试点工作，扩大下放事项，优化机构设置，并在中心镇和市属园区铺开。村级体制改革试点工作稳步推进。基本完成集体林权改革。深化行政审批制度改革。开展第四轮清理工作，调整审批事项232项，精简32%。积极推进并联审批、网上审批和电子监察。深化户籍制度改革。实行积分制入户和居住证制度，近1.2万名新莞人获得积分入户资格。深化公共事业改革。推动医疗卫生事业改革，建立基本药物制度，确立4家公立医院改革试点。认真履行部省协议，推进民政工作改革，推行居家养老服务，开展社会工作试点。完成水务一体化管理以及农村公路养护改革。深化机关作风建设。加强廉政和执行力建设，将加工贸易转型等13项重要工作和十件实事纳入行政问责范围。落实重大行政决策程序和政务公开制度，市长办公会向媒体开放。深入开展财政绩效评价。实施大规模干部培训。继续开展市民评机关活动。深化区域合作发展。推进深莞惠一体化发展，制定“四年大发展”工作方案，三市在界河污染共治、交通对接、社会管理联动和社保卡互通等方面取得了积极进展。落实“双转移”战略，市财政投入1亿元推进莞韶、莞惠产业转移园区建设。投入援建资金9亿元，提前完成援建四川映秀任务。制定支援新疆农三师图木舒克市总体方案和规划，3个试点项目竣工。落实推进与云浮、韶关两市“双到”扶贫开发，筹集帮扶资金2.2亿元，帮助50.8%的贫困户实现了脱贫。捐赠甘肃玉树灾区6311万元，援助广西河池、西藏林芝、新疆伊吾等对口帮扶地区财物1986万元。

刚刚过去的2010年，也是实施“十一五”规划的最后一年。五年来，我们积极稳妥地推进经济社会双转型，较好地完成了“十一五”各项目标任务，经济社会发展取得了新的成就。

这五年，是综合实力稳步增强的五年。我们在极其困难的情况下，努力实现了高基数上的平稳较快发展。全市生产总值五年增加2000亿元，财政总收入增长1.3倍，年度固定资产投资、社会零售总额均突破1000亿元，各项存款余额突破6000亿元，出口总额稳居全国第四。镇街平均可支配收入达到8.7亿元，村组平均可支配收入超3000万元。东莞被列为全国改革开放18个典型地区之一。

这五年，是结构调整逐步加速的五年。我们解放思想，科学谋划，以深入发动强化共识，以激励政策明确导向，以罕见力度稳企扶企，以危机倒逼助推转型，结构调整明显加快。呈现出三大产业结构、内外经济结构、企业经营形态、产品市场结构优化，以及人均经济指标、自主创新能力、节能减排水平、产业集聚程度提升的良好态势。工业技改投资五年增长4倍，专利授权量跃居全省第二。

这五年，是城乡环境持续优化的五年。我们加强统筹，以城带乡，全社会投入2300多亿元推进城市建设，投入200多亿元治理水污染，建成数百宗工程防灾减灾，扎实推进“四清理”、“五整治”以及森林公园建设。城乡一体化程度更高，市中心区地位更加凸显，镇村形象更加靓丽，水环境明显好转，空气质量持续改善。先后获得了全国绿化模范城市、国际花园城市、国家园林城市等荣誉。

这五年，是社会管理全面提升的五年。我们强力打好“治摩”、“治吧”、整治出租屋、扫除黑诊所等战役，全面推进消防安全大排查、大整改、大培训、大建设，坚持开展民间纠纷调处、劳动争议调解、市镇领导接访等行动，建立健全维稳综治、安全生产、应急管理长效机制。各类事故指标、刑事案件和信访数量稳步下降，群众安全感明显增强，社会管理水平不断提升。东莞获评全国社会治安综合治理优秀市。

这五年，是群众得到更多实惠的五年。我们始终坚持民生为本，市财政累计投入453.4亿元用于发展社会事业，公办义务教育实现了全免费，教育强镇和社区医疗卫生服务实现了全覆盖，基本医疗和养老保险实现了城乡统筹，公园和博物馆等实现了免费开放。城市居民和农民收入稳步增长，最低工资和最低生活保障标准逐步提高。城乡公共服务更加完善，市民素质不断提升。东莞进入全国文明城市行列。

各位代表！

五年的发展令人鼓舞，奋斗的历程让人铭记。五年风雨兼程，五年春华秋实，我们在困难中保持了平稳发展，在危机中加快了转型步伐，在磨砺中经受了严峻考验，在奋进中坚定了信心决心，在实干中铸就了新的辉煌。这离不开上级和市委的坚强领导，离不开市人大、市政协与各民主党派的监督支持，离不开广大市民、新莞人和外来投资者以及驻莞部队的共同努力，离不开港澳台同胞、海外侨胞以及国际友人的关心帮助。在此，我们表示衷心的感谢！

五年的发展启示良多，积累的经验弥足珍贵。我们深切地体会到：必须强化转型才有出路的共识。只有深入践行科学发展观，切实转变发展方式，推动经济社会双转型，才能增强核心竞争力和抗风险能力。必须坚定制造业立市的方向。实体经济是持续发展的重要支撑，制造业是东莞经济的品牌和特色。只有坚定不移地建设现代制造业名城，才能强化产业依托，发挥比较优势。必须坚守强镇才能强市的理念。镇村是东莞的发展之基、实力所在、惠民所依，只有坚持不懈地做强镇域经济，才能筑牢全市发展根基。必须坚持实施环境取胜的战略。城乡一体、组团发展、绿色宽畅是东莞城市环境的独特优势，只有坚定生态宜居的发展定位，深入实施环境取胜战略，才能赢得主动。必须发扬实干创新发展的精神。实干铸就了东莞“海纳百川、厚德务实”的城市精神，培育了东莞人开拓创新的胆识和勇气，成就了东莞经济社会发展的领先地位。只有发扬实干精神，永不止步，永不懈怠，才能永葆活力，开创未来。

五年的发展攻坚克难，当前的问题仍有不少。我们清醒地看到：经济回升基础仍不牢固。尽管已基本走出金融危机的低谷，但部分经济指标与危机前比，与珠三角其他城市比，还存在一定差距。结构调整力度仍需加大。外向依赖、资源依赖程度还比较高，优质品牌企业还不够多、不够强，企业总体技术水平和自主创新能力有待进一步提升。环境资源压力仍然较大。土地、能源等资源制约突出，污染治理与环境保护力度仍需加大。均衡发展水平仍待提升。镇村之间发展不平衡，村组集体收不抵支现象有所增多。社会管理工作仍需加强。社会不稳定因素依然较多，治安管理、安全生产、信访矛盾、教育公平、医疗改革等仍然面临较大压力，近来物价的持续上涨也给群众生活造成了一定影响。政府服务效率仍待提高。机关作风有待深入改进，办事难等依然是群众反映强烈的问题之一。对此，我们将高度重视，积极应对，努力破解。

“十二五”时期奋斗目标和主要任务

“十二五”时期将是世界经济格局的复杂变革期，是全球新兴产业、新兴市场孕育发展的重大机遇期，是我国加快转变经济发展方式的攻坚期。对东莞来讲，这将是深入推进双转型、加快现代化建设的重要五年。虽然我们面临着外向依存度过高、先发优势弱化、企业成本上升、节能减排和人民币升值压力增大等挑战，中央货币政策收紧、积极财政政策力度减弱以及国内较大的通胀压力等，也将对东莞经济带来一定的影响。但与此同时，我们也拥有许多有利条件：外部经济环境有望逐步好转，我国城镇化建设加快，内需拉动效果逐步显现；全市发展基础扎实，政府和民间的创造力较强，推动转型发展的思想更加统一、目标更加明确、方法更加对头，东莞和谐的人文环境、舒适的城市环境、公平的市场环境以及完备的产业体系仍然受到客商信赖。因此，我们必须准确把握国内外的形势变化，增强机遇意识、忧患意识和创新意识，既要对困难有充分的估计和应对的准备，又要坚定信心决心，扬长避短，主动作为，努力推动东莞经济社会沿着科学发展的道路稳步前进。

“十二五”时期东莞发展的总体要求是，以科学发展为主题，以加快转变经济发展方式为主线，以“加快转型升级，建设幸福东莞”为核心，以产业结构调整升级和城市升级为重点，深入推进经济社会双转型，努力当好推动科学发展、促进社会和谐的排头兵。主要发展目标：生产总值年均增长8%，结构调整取得明显突破，城市软实力明显提升，体制机制明显完善，人民幸福感明显增强。

要实现上述目标，必须处理好发展速度与发展方式、硬实力与软实力、经济增长与民生福祉、政府与市场、改革发展与稳定的关系。必须坚持先行先试、转型发展，坚持调整结构、创新发展，坚持节能减排、绿色发展，坚持统筹协调、和谐发展，坚持以人为本、惠民发展，坚持扩大开放、合作发展。要重点在以下四大方面下功夫：

一、深入提升“东莞制造”品牌。毫不动摇加快产业结构调整，推动经济发展方式从资源主导、粗放增长向创新主导、绿色低碳转变，从多轮驱动、零散发展向园区带动、集群发展转变，从外向依赖向内外并重转变。大力调整产业存量，深入开展全国加工贸易转型升级试点，改造提升传统产业，加大扶优扶强力度，促进现有企业做大做强。大力提升产业增量，提高利用内外资水平，实施战略性新兴产业五年倍增计划，努力实现以大补小、以优代劣。大力提高产业质量，加强科技创新和品牌建设，壮大民营经济，扩大内需市场，落实节能减排，实现高端化、本土化、集约化发展。大力强化产业服务，加快现代服务业发展，建设金融强市、珠三角新兴物流城市、会展休闲之都。大力优化产业布局，加快形成“一个核心区、三大经济带、五大产业集聚区”的产业空间布局，打造更多更高层次的产业集群。全面构建起先进制造业实力较强、现代服务业支撑有力、都市农业高效集约的现代产业体系，切实提高核心竞争力和抗风险能力。

二、深入打造生态宜居城市。对城市总体规划进行修编，按照“强核、宜居、内畅、外联”的思路，进一步完善城市格局、提升城市品位、塑造城市性格。坚持基础设施先行，以构建内聚外联的轨道交通为突破，完善一环六横六纵三连的高快速路网、四通八达的主干支次路网和便捷顺畅的公交体系，推动市民出行方式转变，建成市内半小时生活圈；以打造“智慧东莞”为方向，推进电信网、广播电视网、互联网三网融合，加快物联网发展，完善城乡全覆盖的水电气设施和通讯网络。坚持组团发展，大力提升以中央商务区、商贸区、生态休闲区为重点的市中心区建设，推进各镇标志性片区和成熟社区的建设，力争把主城区建设成为城市形象展示区、高级人才集聚区和成熟社区示范区，各个镇建设成为具有“小市富县”配套水平的城市支点。坚持环境取胜战略，突出以绿为魂、水气同治，深入推进环境整治，努力做到天更蓝、水更清、城更绿。坚持建管并重，大力推进城市管理精细化、数字化、社会化。努力争创国家生态城市。

三、深入推进文化名城建设。紧紧把握住提高人的素质这个核心要求，深化精神文明建设，发展文化教育事业，全面提升城市软实力。培育现代城市文明，弘扬“海纳百川、厚德务实”的城市精神，加强思想道德建设，建设学习型城市，形成良好社会风尚，践行社会主义核心价值观，巩固全国文明城市地位。加快教育事业改革发展，实施学前教育三年行动计划，推动义务教育优质均衡发展，提高普通高中教育水平，打造职业教育品牌特色，促进高等教育跨越发展、成人教育多元发展，努力建设全省推进教育现代化先进市、珠三角重要人力资源培训配置基地和人才高地。推进文化名城建设，打响东莞特色文化品牌，促进文化事业产业繁荣发展，初步建成全国公共文化服务名城、国家历史文化名城、全国现代文化产业名城、岭南文化精品名城。

四、深入建设和谐幸福家园。坚持发展民生社会事业，维护安定局面，增进社会和谐，不断提高市民幸福指数。推动公安护民，努力实现社会治安根本性好转，信访数量和安全生产事故逐年降低，预防和处置突发公共事件的能力切实提高，市民安全感显著增强，建成全国和谐劳动关系示范区。推动强镇富民，推动镇村集体经济增资减债，扶持欠发达地区加快发展，打造更多的名镇名村，进一步健全覆盖城乡的就业服务体系，以此为依托促进充分就业和自主创业，实现居民收入增长与经济发展同步。推动卫生健民，建立起比较完善的公共医疗卫生服务体系，看病难、看病贵问题得到明显缓解，人民健康水平不断提高。推动社保惠民，全市居民的参保面不断扩大，人人享有保障的目标基本实现。推动服务便民，建立现代社会工作制度，建成覆盖城乡的养老服务网络，建成10分钟公共服务圈，初步实现基本公共服务均等化。

2011年工作安排

2011年是“十二五”开局之年，做好今年政府工作，意义十分重大。综合考虑各方面因素，2011年全市发展的主要目标是：生产总值增长8.5%，市财政一般预算收入增长10%，固定资产投资总额增长9%、社会消费品零售总额增长14%、外贸出口总额增长8%，城市居民人均可支配收入和农民人均纯收入增长8%，单位生产总值能耗下降2.52%，城镇登记失业率、居民消费价格涨幅分别控制在3%以内和4%左右。

今年政府工作的总体要求是：全面贯彻中央十七届五中全会、中央经济工作会议、省委十届八次全会和市委十二届七次全会精神，坚持以科学发展为主题，以“加快转型升级，建设幸福东莞”为核心，认真落实国家宏观调控政策，着力调整产业结构、转变发展方式，着力增强内需拉动、力促平稳增长，着力加强建设管理、提升发展环境，着力办好社会事业、保障民生改善，巩固和扩大应对金融危机成果，促进经济社会又好又快发展，为“十二五”发展奠定坚实的基础。

围绕以上目标要求，今年要重点抓好以下九方面工作：

一、着力调整产业结构，加快构建现代产业体系

扎实推进产业结构调整，加快转变经济发展方式，不断提升经济发展质量。

加快加工贸易转型。抓好全国加工贸易转型升级试点工作，制定方案，争取政策支持，用好专项资金，加强转型服务，推动来料加工企业转“三资”或民营，支持企业向研发和销售环节拓展。加快发展保税物流，继续举行“莞货全国行”活动，加强与大型零售、电子商务机构的合作。改革加工贸易管理模式，推进外经贸、海关、企业三方联网管理。

加快传统产业改造升级。制定传统产业集群发展和转型升级的指导意见。坚持专业兴镇，扶持各镇集中精力发展1—2个优势传统产业。推广专业镇、专业市场与会展业联动发展模式，加强专业市场整合培育，办好各类知名展会。探索建立工业设计中心。加强“东莞老字号”宣传推介，扶持传统特色企业发展。

加快战略性新兴产业培育。制定战略性新兴产业发展规划及配套政策，重点推动半导体照明、高端新型电子信息、电动汽车、太阳能光伏四大产业发展。加快省市共建产业基地建设，争取电动汽车等产业纳入省级基地。加大新兴产业骨干企业的引进培育，实行用地用电优先保障，政府采购优先考虑，带动形成新兴产业集群。

加快现代服务产业发展。落实新兴物流城市实施意见，推动“一港三带六园区”的规划建设，加快海港航运、城市配送等发展。推进市金融商务区建设，深化区域金融合作，发展新型金融机构，促进地方金融改革创新。加快商业网点规划修编，整合完善西正、东纵、世博、鸿福、华南摩尔等商贸集聚区，提升主城区商业档次。发展旅游、设计、会计、法律等产业。

二、着力加强科技创新，不断提高自主发展能力

整合完善现有政策，五年投入100亿元，深入实施科技东莞工程，提升产业竞争力和自主发展能力。

积极培育“两自”企业。支持企业组建研发中心、重点实验室，资助重大科技专项、粤港招标项目和科技合作交流项目，提升企业技术创新水平。落实税收减免政策，引导企业加强技术改造。深入开展质量强市活动，扶持品牌经营，试行品牌质押，加强区域品牌培育与保护，支持企业参与行业标准制订。

加强创新平台建设。加快建设中科院信息技术研发中心等公共平台，支持半导体照明等行业以及茶山食品、沙田港口物流等专业镇创新平台建设，抓紧建设国家半导体光源产品质检中心、国家城市能源计量中心等检测平台。加强对已建平台的运营管理。落实人才引进政策，积极引进培育科技创新团队和领军人才，建立高层次人才列席重大会议制度。

深化产学研合作。设立产学研合作专项资金，推动科技金融结合，支持技术研发。建设一批产学研示范基地，组建太阳能、云计算、物联网等产学研联盟，加快散裂中子源与民用核技术产业基地建设。实施科技特派员计划，办好科技合作周，推动企业与高校院所交流对接。推进专利培育、知识产权优势企业认定工作，发展知识产权交易市场。

引导扶持民营经济发展。完善和落实扶持民营经济政策措施，选定一批成长型民营企业进行重点帮扶，引导民间资本投向先进制造业领域，加强技术引进消化吸收再创新。发挥行业协会和商会作用，支持兴建市民营大厦。

三、着力实施引优扶强，提升产业集约发展水平

充分发挥园区的产业集聚和辐射带动作用，加力引进和培育优质企业，不断壮大产业实力。

加大园区开发建设力度。加强松山湖招商推介，用好国家级园区优惠政策，完善各项配套，推动落户项目尽快投产。抓好虎门港二期市政工程及麻涌港区建设，建成东莞保税物流中心和虎门港数据中心，提升港口吞吐量。加快生态园基础设施建设，全面开展招商活动。完成长安新区总体规划编制，争取尽快进入开发阶段。制定镇街工业集聚区整合改造指导意见。

加大优质项目引进力度。完善招商引资目录，建立重大项目洽谈、决策快速反应机制，积极引进跨国企业、央企和大型民企。开展日韩台驻点招商，逐步建立政企高层沟通机制。完善用地指标分配机制，优先解决一批优质项目用地问题。加强对镇街招商的协调指导，鼓励合作引进大项目。

加大龙头企业培育力度。落实能源保障、办事优先等政策，大力扶持521家重点工业企业、现代产业体系“四个30项目”、商贸流通业“四个十大”项目、民营工业50强和服务业50强等。选定一批具备潜力的企业进行重点扶持，力争两年内培育主营业务收入超50亿元企业15家以上，超100亿元3家以上，并对实现目标的企业给予奖励。认定第四批上市后备企业，力争新增上市3—5家。

四、着力加快改旧建新，推动城乡环境持续优化

启动城市总体规划修编，以重点项目、“三旧”改造、环保工程等为抓手，深入推进城乡规划建设，进一步扩大投资拉动，完善城市功能，增创环境新优势。

推动交通项目建设全面提速。积极配合穗莞深、穗莞惠城际轨道交通工程，加快市轨道交通R2线建设，加强沿线土地规划统筹。推进东莞新火车站及其配套建设，加快东莞东站升级改造。建成广深沿江高速东莞段一期、虎门港支线一期、东莞大道延长线，加快从莞高速、深圳外环、博深高速、莞番高速东莞段和虎岗高速延长线、环莞快速二期、松山湖大道常平连接线，以及东江梨川、东平大桥等建设。完成中麻公路、S120石排至桥头段、S256南城至虎门段、S358长安至虎门段改造，抓紧石大公路、东部快速企石至桥头段改造，推进沿海公路、疏港大道等建设。

推动城市功能配套加快完善。加快中央商务区、商贸区、生态休闲区的规划建设，抓紧推进南城总部经济片区、东城中心片区、东莞火车站石龙茶山新片区等建设，建成广电中心、市中医院新院、篮球中心等项目，加快工人文化宫、市民艺术中心等项目，进一步增强主城区功能。继续完善城市水电气网络，推进水利防灾减灾工程，建成110千伏及以上输变电工程16项、天然气管网140公里、改造供水管网600公里。加快信息基础设施建设，提升城市信息化水平。

推动“三旧”改造取得突破。用好政策优惠期，加快解决历史用地手续问题。加强改造方向引导，注重开发产业性项目和公益性项目。实行领导挂钩督导，简化工作流程，推行并联审批和逾期同意制，加强政策宣传辅导，加快解决征地、拆迁、审批等难题。抓好试点建设，强化示范带动，推动已批项目加快开工建设。

推动环境整治工程深入实施。严格落实生态控制和耕地保护红线，研究分期实施生态保护区退果还林工程。编制全市绿道网总体规划，启动市级和社区绿道建设。加快黄旗山城市公园的建设和已建森林公园的配套，启动凤岗南门山、清溪山水天地等森林公园建设。加强社区绿化，新建一批乡村公园，创建一批宜居社区。加强污水处理厂运营管理，加快支次截污管网建设，推进运河及挂影洲中心涌综合整治。加快4座垃圾处理厂和7个环保专业基地建设。严格落实节能减排责任，加强排污监控和环境监测，实施清洁空气行动计划，推广节能技术和清洁生产，强化土地执法监察，盘活存量土地。

五、着力扶持镇村发展，切实增强基层活力

镇村是全市发展的重要基础，集体经济是东莞的特色和优势，必须促进镇村持续稳定发展，打造更多的名镇名村。

加力推进市内帮扶。改革市镇财政管理体制，通过提高分成、超收奖励、增加补助等措施，每年增加镇街财力约15亿元。出台激励村一级转型的财政政策。开展市内“双到”扶贫，继续实施原有扶贫贷款贴息、公共管理支出补助等措施，设立定点帮扶资金，延长借款期限，扩大使用范围，对所有基本农田实行生态补偿，实施用地收费返还。年内帮助60%的欠发达村和贫困户脱贫。

理顺镇村管理体制。深入推进简政强镇，推动中心镇和三大市属园区加快改革，用好下放事权。推进黄江、厚街村级体制改革试点。设立农村经济风险储备金，加强镇村集体资产和债权债务管理，出台农村干部薪酬管理办法，制定政经分账核算和集体经济多元化经营的指导意见，严控非生产性支出和超前分配。做好镇村（居）班子和农村股份经济组织换届工作。

发展现代都市农业。加快农业产业园建设，加强项目引进，打造精细农业示范区。推进标准化基本农田建设，促进农业用地集约和规模经营，培育农业龙头企业和专业合作社。开展科技下乡，落实农机购置补贴，推广先进装备，提高生产效益。加快渔区建设改造，稳定海洋渔业发展。

六、着力强化社会管理，维护和谐稳定局面

坚持防范与打击并重、服务与管理并举，不断提高社会管理水平，努力维护社会稳定。

继续狠抓社会治安和安全生产。保持高压态势，加强治安复杂场所整治，严厉打击恶性犯罪和多发性犯罪，突出打击汽车抢劫、入室盗窃、黄赌毒等。推进“平安公交”建设，加强出租屋管理。探索推动巡警体制改革，强化公安信息化和执法规范化建设。加大安全宣教和隐患排查整治力度，实施消防“防火墙”工程，加快“三镇一港”化工园区建设。完善各级应急机构建设，建成市安全生产应急救援指挥中心，提高突发事件联合处置能力。

继续狠抓劳动监察执法和信访调解。加强人文关怀，改善用工环境。开展劳动监察网格化和网络化建设，提高劳动仲裁处理效率，严厉打击欠薪逃匿等行为。建成市人民来访接待厅，完善基层综治信访维稳机构，强化矛盾排查化解，落实领导接访包案和干部下访制度，妥善解决群众合理诉求。

继续狠抓市政交通和综合执法管理。增加公交运力和线路，加快完善公交站点和公共停车场建设，提升公交服务质量。加强交通疏导和执法值勤，加快交通拥堵点的改造，完善步行系统、自行车道以及视频监控、护栏设施。加强城乡内涝整治，修缮农村巷道和排污排水设施。探索生活垃圾分类收集处理，继续改进环卫保洁工作。加强城市管理综合执法，深入整治城市“六乱”，抓好流动商贩管理和违法建筑整治。

继续狠抓平安和谐社区建设。启动“幸福家园”社区服务体系建设，实施社区工作人员培训工程，打造社区优质服务示范点，继续抓好省“六好”平安和谐社区、市平安社区创建工作。加大社会工作力度，完善相关配套政策，建立政府购买公共服务制度。加快成熟社区建设，出台考核验收标准，推动社区功能配套加快完善。

七、着力发展各项事业，有效保障民生改善

坚持以人为本，大力改善民生，继续办好十件实事，推动社会事业发展，促进社会全面进步。

铺开文化名城建设。设立5年共50亿元的专项资金，出台配套政策。推进全国公共文化服务示范区建设，完善城乡文化设施，实施文化惠民工程。启动国家历史文化名城创建，加强非物质文化遗产保护，建设中国近代史主题公园。加快印刷、创意设计、现代传媒等行业发展。打造具有岭南文化特色的艺术精品。实施城市形象塑造和提升工程。力保蝉联全国文明城市。

提升教育发展水平。增加财政对教育的投入，提高公办中小学的生均经费标准。加大市镇财政对学前教育的支持力度，壮大公办幼儿园规模，提升民办发展水平，落实村（社区）和新建小区办园，提高幼师待遇，规范学前教育管理。完成10所高中学校新建扩建和职教城一期工程，落实省职业教育综合改革试验区各项工作，支持高等教育发展。

推进健康城市建设。深化医药卫生体制改革，完善基本医疗保障制度，实施基本药物制度，稳妥开展公立医院改革试点。健全基层医疗卫生服务体系，完善社区卫生服务功能，提高服务质量。加强重大传染病和职业病防控。强化食品药品安全监管，加大检测抽查力度。完善各项体育设施，推动群众体育和竞技体育蓬勃发展。继续做好人口计生工作。

做好就业社保等工作。鼓励开办“村民车间”，实施青年见习培训计划，开展农民工专场招聘，帮助市民充分就业创业。完善城乡一体社保体系，提高离退休人员基本养老金，将低保标准从400元提高到440元，实施低保动态管理，增加特困低保家庭生活补助。制定临时救助办法，探索为困难人群购买重大疾病保险，完善专项慈善基金运行机制。落实积分制入户和居住证制度，探索基本公共服务积分制管理。抓好“菜篮子”工程，加强物价管理，向困难群众发放临时物价补贴。加强住房保障，建设雅园新村二期。扎实开展双拥共建，争创全国双拥模范城“七连冠”。继续做好国防人防、科普法普、外事侨务、工青妇幼、民族宗教、统计审计、档案方志、气象、打私等工作。

八、着力落实规划纲要，积极扩大开放合作

深入实施珠三角规划纲要，积极落实内地与港澳台相关经贸合作协议，落实对口帮扶任务，推动区域合作与协调发展。

强化深莞惠一体化合作。共同编制深莞惠区域协调发展总体规划，论证建立产业合作示范区。加快跨界路网对接，优化跨界公交线路，推进车辆通行年票互通。加强界河联合整治，开展大气污染联合防治。加强水资源开发利用、流动人口服务管理、打击跨区域犯罪和处置突发事件的协作。推进职称鉴定和证书互认互通、医疗保险异地结算、养老保险无障碍转移，以及劳动监察、劳动仲裁合作。

强化与港澳台等交流合作。加强与香港、澳门在城市管理、金融、会展、旅游、教育、卫生等方面的合作，落实产业服务制度，促进在莞港资企业转型升级。加强与台湾高科技产业、现代农业等合作，推动松山湖台湾高科技园尽快形成规模效益，推进虎门港与高雄港、基隆港的合作。加强与东盟合作，开拓新兴市场。

强化对口援建与扶贫协作。落实援疆、援藏计划，抓紧启动一批民生项目。扎实推进与云浮、韶关两市“双到”扶贫开发，力争提前实现80%贫困户脱贫的目标。用好省市扶持资金，加强莞韶、莞惠产业转移园基础设施建设和招商引资。完成援建汶川映秀收尾工作。继续做好对广西河池等地的对口帮扶。

九、着力加强自身建设，打造廉洁高效政府

坚持依法行政，优化政府服务，狠抓工作落实，提高政府公信力和执行力。

进一步加强依法行政。认真执行人大决议，主动接受社会监督。落实重大行政决策程序规定，健全行政决策合法性审查机制，促进科学民主决策。制定行政处罚裁量标准，完善处罚程序，开展执法评议考核，进一步规范执法行为。推进电子政务建设，完善网络问政平台及政务信息共享平台。推动中心镇成立法制机构，提高依法行政水平。

进一步提升服务效能。深化行政审批制度改革，完善和落实重点项目并联审批、超时默认制度，推动一批群众常办事务上网审批。完善政府采购实施办法，加强财政资金绩效评价，全面推行市级财务核算信息集中监管改革，提高资金使用效率。推广“一站式服务”，严格执行首问责任、限时办结、责任追究等六项制度。加快事业单位分类改革。落实各项扶企制度，帮助解决好用工、用电、用地、融资等问题。

进一步改进机关作风。加强重大决策和重点工作的跟踪督查，落实现场办公和季度片会制度，促进难点问题解决。严格厉行节约，严控会议和庆典活动，建设全市视频会议系统。落实《廉政准则》，推进廉政建设和反腐败斗争，建设综合型纪检监察平台。健全明查暗访机制，坚决纠正不正之风。

各位代表！“十二五”的蓝图已经绘就，各项目标任务已经明确。站在新的起点上，我们必须恪守主抓执行的职责，拿出只争朝夕的干劲、不甘人后的拼劲，在抓落实上见分晓、比高低；必须牢记执政为民的宗旨，坚持一切为了群众，一切依靠群众，在惠民生上办实事、办好事；必须提振奋发有为的精神，坚定改革创新的决心，强化攻坚克难的锐气，在促转型上求突破、勇争先，为建设幸福东莞而努力奋斗！
